# Identification of stroke-associated-antigens via screening of recombinant proteins from the human expression cDNA library (SEREX)

**DOI:** 10.1186/s12967-015-0393-4

**Published:** 2015-02-22

**Authors:** Toshio Machida, Motoo Kubota, Eiichi Kobayashi, Yasuo Iwadate, Naokatsu Saeki, Akira Yamaura, Fumio Nomura, Masaki Takiguchi, Takaki Hiwasa

**Affiliations:** Departments of Neurosurgery, Chiba Cardiovascular Center, Ichihara, Chiba Japan; Department of Neurosurgery, Kameda Medical Center, Chiba, Japan; Department of Neurological Surgery, Graduate School of Medicine, Chiba University, Chiba, Japan; Chiba Prefectural University of Health Sciences, Chiba, Japan; Department of Molecular Diagnosis, Graduate School of Medicine, Chiba University, Chuo-ku, Chiba 260-8670 Japan; Department of Biochemistry and Genetics, Graduate School of Medicine, Chiba University, Chiba, Japan

**Keywords:** Stroke, SEREX, Antibody biomarker, Atherosclerosis

## Abstract

**Background:**

Because circulating antibodies against a variety of antigens have been detected in patients with coronary heart disease, carotid atherosclerosis and those who have suffered a stroke, it is suspected that immune response may be one of the mechanisms of atherogenesis The objective of this study is to identify novel antibodies in ischemic stroke patients by screening the expressed recombinant proteins using a human cDNA library (SEREX).

**Methods:**

To identify the candidate antigens, cDNA library was screened by SEREX using plasma from ten patients with ischemic stroke. Subsequently, via ELISA using recombinant proteins and synthetic peptides, the serum antibody levels were measured in two independent patient/healthy donor (HD) cohorts (142 and 78 in the 2nd screening and a validation cohort, respectively).

**Results:**

The initial screening resulted in the identification of six candidate antigens. Of these antigens, replication protein A2 (RPA2) was determined to be the antigen associated with stroke (P < 0.05) by ELISA with 2nd screening and validation cohort. Multifactorial logistic regression analysis showed that the increased levels of the RPA2 antibodies (RPA2-Abs) associated with stroke independent of other risk factors for stroke (P < 0.05). Receiver operating curve analysis demonstrated that the area under the curve from ELISA using GST fusion RPA2 and synthetic peptides (bRPA2-132) were 0.867 (95% CI: 0.798-0.936) and 0.971 (95% CI: 0.940-1.00), respectively. If the cut-off value of the bRPA2-132-Ab level was determined to be 0.334, the sensitivity and specificity of the antibody level as the diagnostic marker for stroke were 0.323 (95% CI: 0.209-0.453) and 1.00 (95% CI: 0.713-1.00), respectively.

**Conclusions:**

SEREX identified RPA2 as the antigen associated with ischemic stroke and serum auto-antibodies against RPA2 elevates in stroke patients. RPA2-Abs could become a biomarker for the evaluation of ischemic stroke at risk.

## Background

Current studies recognize the role of inflammation in all stages of atherosclerosis and, subsequently, the immune system has become a subject of focus in this field [1]. Both innate and adoptive immunities, which recognize endogenous antigens such as proteins, carbohydrates, lipids and nucleic acids, influence the progression of atherosclerosis [2]. The proposed antigenic proteins that evoke immune responses and affect this progression include oxidized-low density lipoprotein (oxLDL), phosphorylcholine, heat shock proteins (Hsps), apo-A1 and phospholipids as well as the antibodies for these antigens, all of which have been found in elevated levels in patients diagnosed with a cardiovascular disease [3]. Ischemic stroke is the major cause of worldwide mortality and morbidity and one of the primary mechanisms of stroke is atherosclerosis. Along with other cardiovascular diseases, the increased antibody levels for antigens such as Hsp 27, 65 and 70 have been reported in patients with ischemic stroke [3].

Serological identification of antigens by recombinant cDNA expression cloning (SEREX) is an approach to antigenic protein identification that enables large-scale screening [4]. Although originally developed to screen cancer-associated antigens, this method has also been applied to vascular conditions such as transplant-associated coronary artery disease [5], Kawasaki disease [6], and moyamoya disease [7]. To our knowledge, stroke-associated antigens have not been screened for. Based on the notion that antigens other than Hsps are implicated in stroke, we screened a human microvascular endothelial cell cDNA library using plasma from a stroke patient.

## Methods

### Human subjects

This study is as a part of the Genomic and Proteomic Research for Stroke Study at Chiba University Graduate School of Medicine. The Local Ethical Review Board of Chiba University Graduate School of Medicine approved the study and written informed consent was obtained from each subject. The inclusion criteria of the patients is those who suffered ischemic stroke and admitted to three participant hospitals within two weeks from the stroke onset. Healthy donors (HDs) were collected from those who undergo medical checkup and all of them were without history of ischemic stroke. Those with an autoimmune disease were excluded from this study. The population of this study consists of three independent patient/HD cohorts: a 1st, a 2nd and validation cohort (Table 1). Ten patients with ischemic stroke due to severe (>90%) carotid stenosis were selected for the 1st screening cohort and remaining 142 and 78 patient/HD were allocated to 2nd screening and validation cohort, respectively. In order to discuss the relations between the antibody levels and the severity of atherosclerosis, those with known carotid stenosis were preferentially allocated to 2nd screening cohort and thus, among 154 patients in the two cohorts, 37/102 (30.4%) and 1/26 (3.84%) had significant (>50%) carotid stenosis in the 2nd and validation screening cohort, respectively.

**Table 1:** **Baseline characteristics of subjects in 1st**, **2nd and validation cohort**

	**1st Screening**	**2nd Screening**	**Validation**		
	**Patients (n = 10)**	**Patients (n = 134)**	**HD (n = 24)**	**Patients (n = 70)**	**HD (n = 42)**
Age	72.4 (3.53)	68.1 (10.4)	64.4 (8.03)	68.6 (10.3)	53.0 (4.57)
Male gender	9 (90)	106 (79.3)	21 (87.5)	54 (77.1)	25 (59.5)
Hypertension	6 (60)	88 (65.9)	1 (4.17)	45 (64.3)	N/A
Diabetes	3 (30)	35 (25.9)	3 (12.5)	17 (24.3)	N/A
Hyperlipidemia	3 (30)	36 (26.7)	1 (4.17)	22 (31.4)	N/A
Smoking	1 (10)	46 (34.1)	6 (25.0)	24 (34.3)	N/A
Stroke	10 (100)	129 (96.3)	0 (0)	67 (95.7)	0
Stroke etiology			-		-
LAA	10 (100)	34 (21.5)		29 (41.4)	
SVD	0 (0)	62 (39.2)		18 (25.7)4	
CE	0 (0)	14 (8.9)		(5.71)	
UD and others	0 (0)	24 (15.2)		16 (23.9)	
Carotid stenosis >50%	10 (100)	31/102 (30.4)	N/A	38/60 (63.3)	N/A

Baseline characteristics of subjects in 1st, 2nd and validation cohort are displayed. Data represents means (SD) for numerical data and n (%) for categorical data. CE, cardioembolism. HD, healthy donor. N/A, not assessed. LAA, large artery atherosclerosis. SVD, small vessel disease. UD, undetermined ethiology.

### Blood sampling and purification

Blood samples were collected both with and without EDTA from each patient upon admission, centrifuged at 3000 × g for 10 min at room temperature and the supernatant was stored at −80°C. Plasma and serum were used in the screening of the 1st and the 2nd/validation cohorts, respectively.

### Clinical data

Classical risk factors for atherosclerosis, including gender, age, incidence of hypertension, diabetes, hyperlipidemia, and cigarette smoking, were evaluated from clinical records. Hypertension was defined as a history of blood pressure above 140 mmHg in systolic or 90 mmHg in diastolic pressure or the use of antihypertensive agents. Diabetes was defined as having undergone antidiabetic therapy or having a history of diabetes. Hyperlipidemia was defined as having a history of total cholesterol > 220 mg/dl or triglyceride > 150 mg/dl or the use of lipid lowering drugs. Patients were considered smokers if they currently smoked or had a history of smoking. Angiographic stenosis rates were determined in accordance with the measurements of the North American Symptomatic Carotid Endarterectomy Trial (NASCET) [8] and patients were categorized as having significant carotid stenosis when the blockage exceeded 50% by NASCET measurement. The stroke subtype for each patient was also determined according to the criteria of the Trial of Org 10172 in Acute Stroke Treatment classification system [9].

### SEREX screening of cDNA libraries

Recombinant DNA studies were performed with the official permission of the Chiba University Graduate School of Medicine and were carried out in accordance with the rules of the Japanese government. We used a commercially available human microvascular endothelial cell cDNA library (Uni-ZAP XR Premade Library, Stratagene, La Jolla, CA) to screen for clones that were immunoreactive against plasma from patients with severe carotid stenosis. The screening method used in this study is a modification of a published procedure as described previously [10-12]. *Escherichia coli* XL1-Blue MRF’ was infected with Uni-ZAP XR phage and the expression of resident cDNA clones was induced after blotting the infected bacteria onto NitroBind nitrocellulose membranes (Osmonics, Minnetonka, MN) that had been treated with 10 mM isopropyl-β-D-thiogalactoside (Wako Pure Chemicals, Osaka, Japan) for 30 min. The membranes with bacterial proteins were washed 3 times with TBS-T [20 mM Tris–HCl (pH 7.5), 0.15 M NaCl and 0.05% Tween-20], and non-specific binding was blocked by incubation with 1% protease-free BSA (Wako Pure Chemicals) in TBS-T for 1 h. The membranes were exposed to 1:2000-diluted plasma for 1 h. The pre-absorption of plasma antibodies against bacterial proteins is a routine procedure [4]. However, because cross-reactive antibodies to bacterial and human proteins are possible antigens in the case of atherosclerosis, we screened the samples without pre-absorption. After 3 washes with TBS-T, the membranes were incubated for 1 h with 1:5000-diluted alkaline phosphatase-conjugated goat anti-human IgG (Jackson ImmunResearch Laboratories, West Grove, PA). Positive reactions were developed using 100 mM Tris–HCl (pH 9.5), containing 100 mM NaCl, 5 mM MgCl_2_, 0.15 mg/ml of 5-bromo-4-chloro-3-indolylphosphate and 0.3 mg/ml of nitro blue tetrazolium (Wako Pure Chemicals). Positive clones were re-cloned two additional times in order to obtain monoclonality.

### Sequence analysis of identified antigens

Monoclonal phage cDNA clones were converted to pBluescript phagemids by excision *in vivo* using the ExAssist helper phage (Stratagene). Plasmid DNA was obtained from the *E. coli* SOLR strain after transformation by the phagemid. The cDNA insertions were sequenced by dideoxy chain termination using DNA Sequencing BigDye Terminator Kits (Applied Biosystems, Foster City, CA) and an ABI PRISM 3700 DNA Analyzer (Applied Biosystems). Sequences were screened for homology with identified genes or proteins within the public sequence database using the NCBI-BLAST algorithm (http://blast.ncbi.nlm.nih.gov/Blast.cgi).

### Expression and purification of antigenic GST-fusion proteins

Recombinant proteins tagged with glutathione-S-transferase (GST) were constructed by recombining the insertion sequences of pBluescript into pGEX-4T (GE Healthcare Life Sciences, Pittsburgh, PA) vector plasmids. The pBluescript plasmids were digested using a combination of EcoRI and XhoI or SmaI and XhoI. The inserted DNA fragments were isolated using GeneElute™ Minus EtBr SPIN COLUMNS (Sigma-Aldrich, St. Louis, MO). The insertion sequences were ligated in frame to SmaI- and XhoI-digested pGEX-4T-1 or EcoRI- and XhoI-digested pGEX-4T-3 using Ligation Convenience Kits (Nippon Gene, Toyama, Japan). The ligation mixtures were used to transform ECOS™ competent *E. coli* JM-109 (Nippon Gene) and appropriate recombinants were confirmed by DNA sequencing. The constructed pGEX recombinants with the correct insertion in the right orientation were then used to transform competent *E. coli* BL21-RIL-codon-plus (Stratagene). The expression of the GST-fusion proteins was induced by treating the transformed *E. coli* with 0.1 mM isopropyl-β-D-thiogalactoside for 3 h. The GST recombinant proteins were purified by glutathione-Sepharose column chromatography according to the manufacturer’s instructions (GE Healthcare Life Sciences) and dialyzed against 1 mM Tris–HCl (pH 7.5) and 1 mM EDTA.

### SDS-PAGE and Western blotting

To confirm the recombinant proteins to be the GST-tagged one that react with autologous plasma, the proteins were lysed in a SDS sample buffer, incubated at 100°C for 3 min, resolved by 12% SDS-polyacrylamide gel electrophoresis (PAGE) and Western blotted. The blotted proteins were probed with an autologous plasma by which the protein was identified or with goat anti-GST polyclonal antibody (Rockland, Gilbertsville, PA). Anti-RPA2 monoclonal antibody (Oncogene Research Products, San Diego, CA) was used to confirm the presence of purified GST-tagged RPA2.

### Quantitative measurement of serum antibodies against purified proteins by ELISA

Ninety-six-well microtiter plates were coated overnight with 50 μl of 1.5 μg/ml of goat anti-GST antibody (Rockland) in a 0.05 M carbonate/bicarbonate buffer (pH 9.6). The plates were blocked with 150 μl of 1% nonfat dry milk in phosphate-buffered saline (PBS) for 1 h, washed 4 times with 0.1% Tween-20 in PBS (PBS-T) and incubated with 50 μl of purified antigens (10 μg/ml) in PBS-T. Plates were left overnight at 4°C. After 4 washes with PBS-T, 50 μl of serum diluted at 1:200 in a blocking buffer were added to each well and incubated for 2 h. Thereafter, the plates were washed 4 times with PBS-T and exposed to 50 μl of horseradish peroxidase-conjugated anti-human IgG Ab (Jackson) diluted at 1:10,000 in blocking buffer for 45 min at room temperature. Plates were then washed 4 times with PBS-T, developed with 100 μl of 400 μg/ml of *o*-phenylene diamine (Sigma-Aldrich) in a 0.1 M phosphate-citrate buffer (pH 4.5) for 20 min and stopped by adding 30 μl of 8 N H_2_SO_4_. Absorbance at 490 nm was determined using a microplate reader (Emax, Molecular Devices, Sunnyvale, CA). Each blood sample was tested in duplicate against GST-antigen fusion protein or control GST alone from a non-recombinant pGEX vector. GST-tagged WD repeat 36 protein diluted at 1:200, 1:400, 1:800 and 1:1600 versus patient No. 8 was the standard in each plate. After normalization by the standards, the levels of serum antibody were calculated by subtracting the absorbance of serum against GST from that against GST-tagged antigens as described previously [11].

### Peptide synthesis and ELISA

Three epitope sites in RPA2 protein were predicted using the program ProPred (http://www.imtech.res.in/raghava/propred/). Three N-terminal biotinylated peptides were synthesized and used as antigens to examine the antibody levels by ELISA. The amino acid sequence of each peptide was as follows:

- bRPA2-74; biotin-VTIVGIIRHAEKAPT (amino acids between positions 74 and 88)
- bRPA2-90; biotin-VYKIDDMTAAPM (amino acids between positions 90 and 102)
- bRPA2-132; biotin-LRSFQNKKSLVAFKI (amino acids between positions 132 and 146)

The purities of bRPA2-74, bRPA2-90 and bRPA2-132 were 96%, 98% and 93%, respectively. The ELISA plates were pretreated with 10 μg/ml of streptavidin in a 0.05 M carbonate/bicarbonate buffer (pH 9.6). After blocking, the plates were incubated with 50 μl of the peptides (100 μg/ml) in PBS-T.

### Immunohistochemistry

Tissue samples were obtained from surgically resected carotid atherosclerotic plaque. The samples were fixed with formalin and embedded in paraffin. The samples were pretreated by heating them in a microwave in a citrate buffer for 10 min at 500 W. The first antibodies used were the monoclonal anti-human RPA2 antibody (Santa Cruz Biotechnology), the anti-CD68 antibody (Santa Cruz) and the anti-SMC (smooth muscle cells) antibody (Santa Cruz) at a dilution of 1:100. After incubation at 37°C for 60 min, the specimens were incubated with biotin-labeled rabbit anti-mouse-IgG secondary antibody and, next, with streptavidin-labeled peroxidase. Sections were counter-stained with hematoxylin after the DAB reaction as described in the literature [12].

### Statistical analysis

Data were processed using EZR software [13]. Differences between numerical and categorical data with respect to each group were assessed using the Mann–Whitney U or Kruskal-Wallis test and the chi-square test, respectively. Correlation between the numerical data was assessed by linear regression analysis. Multivariate logistic regression analysis was used to find a set of variables classifying the subjects into those with and without a history of stroke. The predictive value of RPA2-Abs for stroke was assessed by receiver operating curve (ROC) analysis and the cut-off values of RPA2-Ab levels were set at the values that maximize the sums of the sensitivity and specificity. All tests were two-tailed and a *P* value below 0.05 was considered significant.

## Results

### Primary screening by SEREX

**Figure 1:**
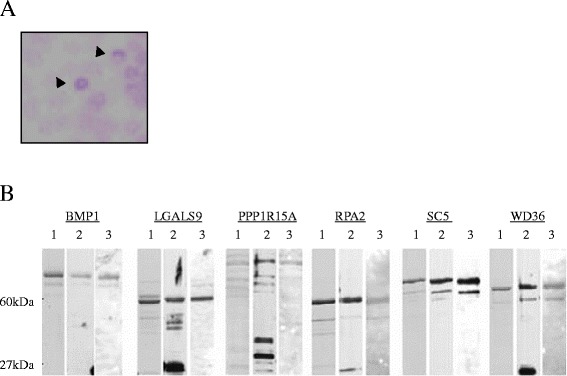
**1st screening by SEREX and Western blot analysis.** Recombinant proteins were blotted onto NitroBind nitrocellulose membranes and reacted with patient plasma (arrow head). **(A)** Affinity-purified GST-tagged antigens were separated on 12% SDS-polyacrylamide gels stained with Coomassie staining (lane 1), or Western blotted using anti-GST antibody (lane 2) or autologous sera (lane 3) **(B)**.

We screened 8 × 10^6^ cDNA clones using plasma from ten patients with ischemic stroke caused by severe carotid stenosis (>90%) and isolated 6 reactive clones (Figure 1A, Table 2). DNA sequence analysis and a subsequent homology search of accessible NCBI databases showed that all 6 clones were independent. These clones were further analyzed by Western blotting and ELISA.

**Table 2:** **Genes identified by SEREX**

**Clone name**	**Gene identity**	**Accession No**.
CU-CA-1	BMP1	NM_006129
CU-CA-14	LGALS9	NM_002308
CU-CA-23	PPP1R15A	BC 003067
CU-CA-26	RPA2	NM_002946
CU-CA-28	SC65	BC 007942
CU-CA-35	WD36	NM_139281

Six independent clones were identified by SEREX using plasma from a patient with ischemic stroke caused by severe carotid stenosis (>90%).

### SDS-PAGE and Western blots of purified antigens

To purify the SEREX-identified proteins, the insertion sequences of the 6 pBluescript plasmids were ligated in-frame into GST-tagged expression vectors. We confirmed by sequence analysis that the recombinant pGEX-4T plasmids were properly recombined and GST-tagged recombinant proteins were affinity-purified using glutathione-Sepharose. Purified proteins were resolved by SDS-PAGE and then either directly stained with Coomassie Blue (Figure 1B, lane 1) or Western blotted against the anti-GST antibody (Figure 1B, lane 2) or the autologous plasma antibody (Figure 1B, lane 3). Almost all of the Coomassie-stained bands were recognized by anti-GST antibody (lanes 1 and 2), indicating that they are GST-antigen fusion proteins and the respective degradation products. The molecular weight of the largest product was similar to that predicted by sequencing analysis.

### Secondary screening by ELISA for antibodies to candidate antigens

**Figure 2:**
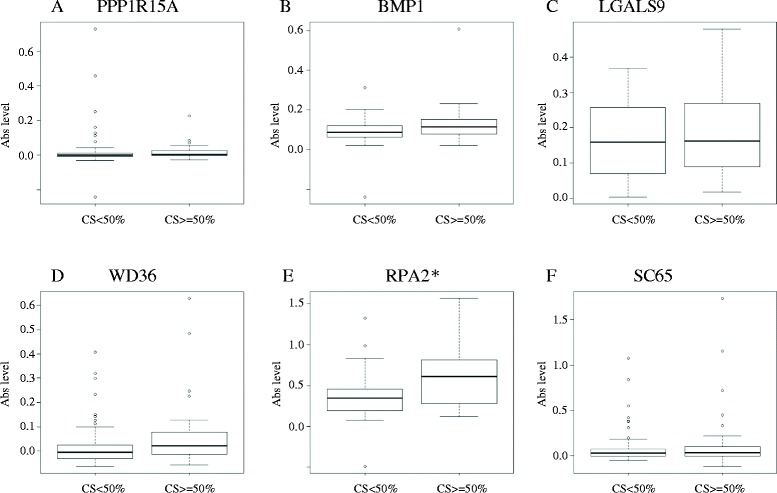
**Second screening by ELISA.** Antibody levels against six candidate antigens were compared between patients with and without >50% carotid stenosis by ELISA **(A-F)**. Only the antibody levels against GST-tagged RPA2 were significantly higher in patients with carotid stenosis **(E)**. *P < 0.05 by Mann–Whitney *U* test. CS, carotid stenosis. Ab, antibody.

**Figure 3:**
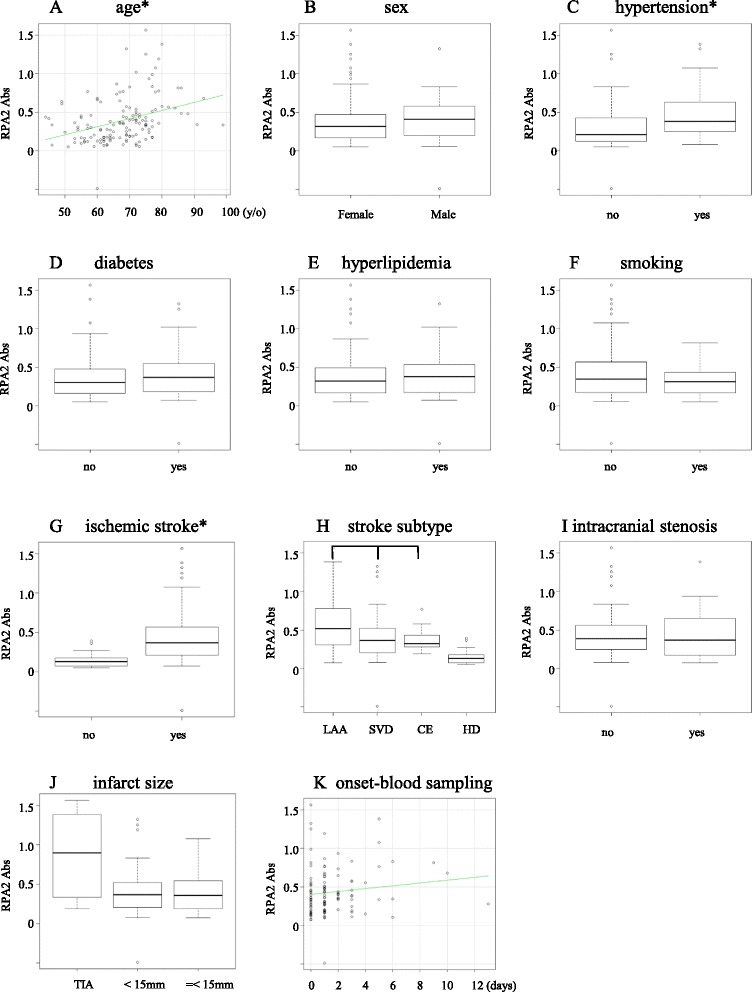
**Association between antibody against RPA2 and other clinical parameters.** The antibody levels against RPA2 weakly correlated with age (*r^2^ = 0.113, P < 0.05). Correlation was examined between RPA2-Abs and age **(A)**, gender **(B)**, hypertention **(C)**, diabetes **(D)**, hyperlipidemia **(E)**, smoking **(F)**, ischemic stroke **(G)**, stroke subtype **(H)**, intracranial stenosis **(I)**, infarct size **(J)** and period between the onset and blood sampling **(K)**. The elevated antibody levels were observed in patients with hypertension and ischemic stroke (*P < 0.05 by Mann–Whitney *U* test). RPA2-Abs, RPA2 antibodies. LAA, large artery atherosclerosis. SVD, small vessel disease. CE, cardioembolism. HD, healthy donor.

**Figure 4:**
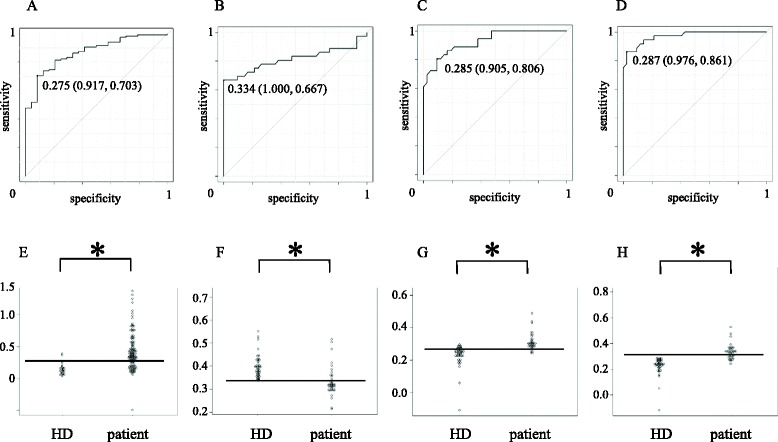
**ROC analysis.** Receiver operating curve (ROC) analysis for the evaluation of diagnostic value of antibodies against purified RPA2 protein **(A)**, bRPA2-4 **(B)**, bRPA2-90 **(C)** and bRPA2-132 **(D)**. Areas under the curve (AUC) were 0.867 (95% CI: 0.798-0.936), 0.812 (95% CI: 0.702-0.813), 0.928 (95% CI: 0.874-0.982) and 0.971 (95% CI: 0.940-1.00), for each antigen. Number in the curves indicate cut-off value of RPA2 Ab level and those in the parentheses indicates specificity (left) and sensitivity (right). Dot plot of antibody levels in patients and healthy donors against purified RPA2 protein **(E)**, bRPA2-4 **(F)**, bRPA2-90 **(G)** and bRPA2-132 **(H)**. The horizontal bar indicates the cut-off value for each antibody level calculated in the ROC analysis. *P < 0.05 by Mann–Whitney *U* test.

To determine whether the six candidate antigens are atherosclerosis-related, we first studied the relations between the antibody levels and the degree of carotid atherosclerosis in the 2nd screening cohort. Of the six candidate antigens, only the antibody against replication protein A2 (RPA2) levels was significantly higher in patients with significant carotid stenosis (≥50%) than those with less advanced lesions (P < 0.05) (Figure 2E), and we further analyzed the relationship between antibodies against RPA2 (RPA2-Ab) levels and other clinical parameters. The RPA2-Ab level is weakly associated with age (r^2^ = 0.113, P < 0.05, Figure 3A) with levels significantly higher in those with hypertension (P < 0.05, Figure 3C). On the other hand, there was no significant difference of the RPA2-Ab levels regarding other parameters including sex, diabetes, hyperlipidemia, smoking, intracranial stenosis, infarct size and timing of blood sampling (Figure 3B, D, E, F, I, J and K). Interestingly, the increases in the antibody levels in stroke patients (Figure 3G) was more prominent than those in patients with carotid stenosis (Figure 2E) and the antibody levels elevate in stroke patients irrespective of the stroke subtype (Figure 3H). These results suggest that the RPA2-Abs associate more strongly with ischemic stroke than atherosclerosis. Therefore, we further analyzed the predictive value of RPA2-Abs for the diagnosis of ischemic stroke. Receiver operating curve (ROC) analysis revealed that the area under the curve (AUC) was 0.867 (95% CI: 0.798-0.936) (Figure 4A). If the cut-off value of the RPA2-Ab level was determined to be 0.275, the sensitivity and specificity of the antibody level for the diagnosis of stroke were 0.703 (95% CI: 0.612-0.784) and 0.917 (95% CI: 0.730-0.990), respectively (Figure 4A and E). Univariate and multivariate logistic regression analysis revealed that, among the other risk factors for stroke, the digitalized RPA2-Ab level was independently and strongly associated with ischemic stroke (O.R.:18.1, 95% CI: 3.10-106, P < 0.01) (Table 3).

**Table 3:** **Logistic regression analysis for the prediction of stroke in the 2nd screening cohort**

	**Univariate**	**Multivariate**				
	**Odds ratio**	**95% CI**	**P**	**Odds ratio**	**95% CI**	**P**
Age (per year)	1.04	1.00-1.09	0.0513	1.05	0.986-1.11	0.138
Male gender	1.63	0.501-7.01	0.604	-	-	-
Hypertension	7.93	2.85-25.8	<0.01	4.17	1.36-12.7	<0.05
Diabetes	1.70	0.570-6.17	0.473	-	-	-
Hyperlipidemia	5.21	1.19-47.55	<0.05	7.47	1.34-41.7	<0.05
Smoking	2.25	0.82- 7.26	0.130	-	-	-
RPA2-Ab*	11.8	4.39-34.9	<0.01	8.16	2.84-23.4	<0.01

*The levels of RPA2-Abs were digitalized by cut-of value of 0.186 in 2nd cohort.

### Validation of elevated p-RPA2-Abs levels in stroke patients using synthetic peptides

To validate the elevated levels of RPA2-Abs in stroke patients and to identify the antigenic epitopes of RPA2, we further examined the RPA2-Abs levels using synthetic peptides in the independent validation cohort (n =78). Three synthetic peptides corresponding to the predicted epitope sites of RPA2 were used as antigens to examine the serum antibody levels. bRPA2-74 with amino acid sequence 74–88 showed lower antibody levels in patients with ischemic stroke as compared with the levels in HD (Figure 4B). On the other hand, bRPA2-90 and bRPA2-132 with amino acid sequences 90–102 and 132–146, respectively, showed significantly higher levels in the patients than those in HD (Figure 4C and D). The predictive values of the antibody levels against the three peptides were evaluated by ROC analysis (Figure 4B and D). The AUCs of bRPA2-74, bRPA2-90 and bRPA2-132 were 0.812 (95% CI: 0.702-0.813), 0.928 (95% CI: 0.874-0.982) and 0.971 (95% CI: 0.940-1.00) for each. If the cut-off value of the bRPA2-132-Ab level was determined to be 0.334, the sensitivity and specificity of RPA2-Ab level for the diagnosis of stroke were 0.323 (95% CI: 0.209-0.453) and 1.00 (95% CI: 0.713-1.00), respectively.

### Immunohistochemistry

**Figure 5:**
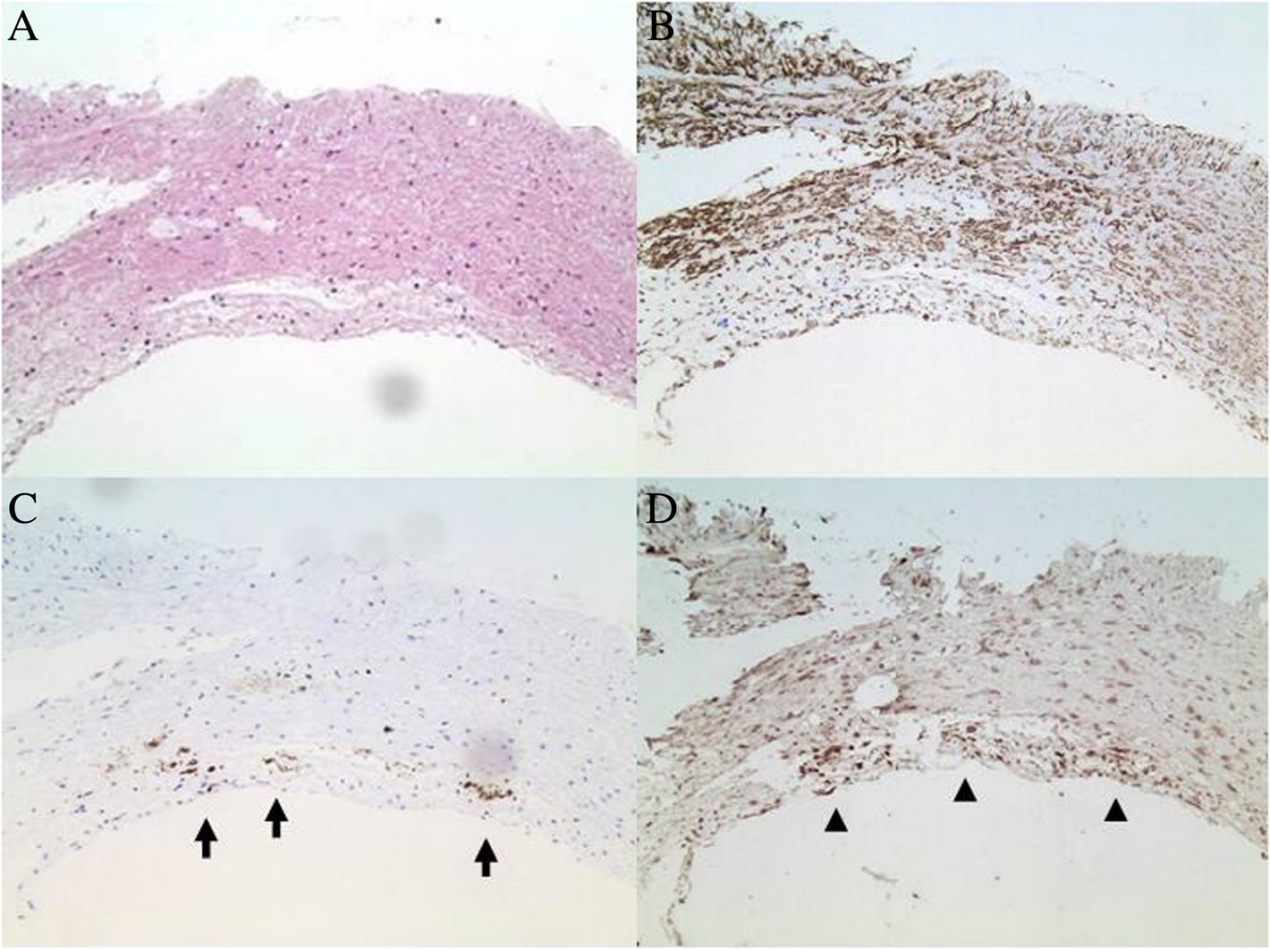
**Immunohistochemistry.** Surgically-excised carotid plaque was stained with hematoxylin only **(A)**, or with anti-SMC **(B)**, anti-CD68 (**C**, arrow) or anti-RPA2 antibody (**D**, arrow head).

We also examined RPA2 protein expression in surgically-resected carotid atherosclerotic plaque using immunohistochemistry. RPA2 was predominantly expressed in the intima of atherosclerotic plaque (Figure 5D). The expression of RPA2 was closely co-localized with CD68-positive macrophages (Figure 5C) but not with smooth muscle cells (Figure 5B).

## Discussion

Much evidence indicates that the immune system is involved in atherogenesis. Atherosclerotic lesions contain significant amounts of macrophages and activated T cells [14-16], suggesting that both innate and adaptive types of immunity are involved in lesion development. Antigens that might activate immune responses are oxLDL, phosphorylcholine, heat shock proteins (Hsps), apo-A1 and phospholipids [3]. Subsequent clinical studies have demonstrated that serum antibodies against these antigens are found in increased quantities in patients with atherosclerotic vascular diseases such as myocardial infarction, carotid atherosclerosis and ischemic stroke and have assessed the role of auto-antibodies as prognostic biomarkers of plaque vulnerability [17-21].

Thus, immune involvement in atherogenesis has been studied in detail, whereas to our knowledge the antigens related to atherosclerosis have not been comprehensively analyzed. Novel antigens might contribute to the diagnosis and the treatment of stroke. We searched for antigens that might be involved in stroke using SEREX, which is a comprehensive method of antigen identification. We isolated 6 cDNA clones in this manner. Subsequent quantitation of antibody levels by ELISA demonstrated that levels of RPA2-Abs were elevated in stroke patients and that these increased levels were independent of the other classical risk factors for atherosclerosis such as age, hypertension, gender, diabetes, hyperlipidemia and cigarette smoking. These results indicate that immunity to RPA2 is a factor associated with the development of advanced atherosclerosis and ischemic stroke.

Immunity to some antigens, such as oxLDL and Hsps, has been assumed to be one of the underlying pathomechanisms of atherogenesis [17,18]. However, the molecular pathomechanisms by which these antigens are implicated in atherogenesis are still not completely understood. The putative scenario is as follows: LDL particles accumulate in the extracellular matrix of atherosclerotic plaque and, following modification by enzymatic degradation and oxidation, they are engulfed by macrophages, presented to CD4^+^ T-cells, thereby activating an adaptive immune response [22]. Antibodies against Hsps of microorganisms, such as *Chlamydia pneumoniae*, might cross-react with human Hsp60 that is overexpressed in the arterial wall by high arterial shear stresses [23,24]. In the case of RPA, whether or not it is implicated in atherogenesis remains to be proved. RPAs are composed of three subunit proteins of 70, 32, and 14 kDa (RPA1, RPA2, and RPA3, respectively). In the rat brain, DNA double-strand breaks (DSBs) were induced by ischemia [25], and BRCA1 which is involved in the repair of DNA DSBs has been shown to be involved in heart function and survival following myocardial infarction [26]. It is noteworthy that the RPA complex works in the upstream pathway of BRCA1 in the DSB repair pathway [27,28]. In atherosclerotic plaque, cells are exposed to reactive oxygen species which can provoke extensive oxidative DNA damage [29,30]. The RPA complex which is required for DSB repair might be expressed in higher amount. In fact, a high rate of expression of the RPA2 protein was observed in the intima of atherosclerotic plaque (Figure 5D). The prominence of RPA here may explain why autoantibody levels are raised against RPA2. It is also likely that antigen proteins leak out from vascular lesion by repeated microenvironmental artery destruction, which frequently happens before the onset of ischemic stroke. Consistently, RPA2 antibody levels were related to ischemic strokes (Figure 3G). RPA2 antigen proteins disappeared rapidly, yet the antibodies can be amplified after repeated exposure to antigen proteins. If we can detect such antibody markers, the onset of stroke can be predicted with a high probability as compared to the predictive powers of the known risk factors alone. Increasing novel antibody markers identified by SEREX other than RPA2 may improve the predictive accuracy for patients with ischemic stroke.

As mentioned above, we hypothesize that the RPA2-Abs might originate from the vascular lesion (i.e. atherosclerotic plaque) and that the RPA2-Abs might become a biomarker for the diagnosis of and, conceivably, for the prediction of stroke. Because all the blood samplings in this study were done after the onset of stroke, when the patients were auto-sensitized to RPA2 is to be proved. However, because IgG antibodies, which we measured in this study, develop several weeks after the exposure to the antigen and because the most of the specimens used in the present study were collected within three days from the onset of stroke, it is plausible that the RPA2-Abs were produced before the onset of ischemic stroke. The divergent antigenic proteins may appear in the surface of the vascular lesion and the IgG antibodies against such newly produced antigens may be produced before the onset of stroke. And therefore, the RPA2-Abs might be a biomarker for the evaluation of stroke at risk rather than the stroke itself. Further study is needed to elucidate the the Ab levels before and after the onset of stroke.

## Conclusions

We have identified six candidate antibody markers for ischemic stroke by SEREX screening. ELISA using recombinant antigen proteins and synthetic peptides revealed that the antibody levels to RPA2 were higher in patients with a history of ischemic stroke than in HD, suggesting that the autoimmunity to RPA2 may play some role in the pathogenesis of ischemic stroke. Further studies are needed to find out whether the elevation of RPA2 Ab could become a biomarker for the evaluation of ischemic stroke at risk.
